# Supplementary material for: Range of chromatin accessibility configurations are permissive of GABAergic fate acquisition in developing mouse brain
Source: BMC Genomics. 2023 Nov 30;24:725. doi: 10.1186/s12864-023-09836-x (PMC10691053; doi:10.1186/s12864-023-09836-x)
Supplement: Supplementary file 1 — Additional file 1: Supplementary Figures and Tables. Supplementary Fig. 1. Distribution of cells in clades and the comparison of the features defined from individual scATAC-seq samples with the joined feature space. Supplementary Fig. 2. Batch effect and quality control analysis of E14.5 scRNA-seq samples. Supplementary Fig. 3. Clustering resolution, cluster reliability and the similarity of labels after the scRNA and scATAC based clustering. Supplementary Fig. 4. Batch effect and quality control analysis of scATAC-seq samples. Supplementary Fig. 5. Analysis of the hierarchical tree of cell clusters. Supplementary Fig. 6. Clustering and neurotransmitter phenotype annotation of E14.5 DI, MB and R1 scRNA-seq data. Supplementary Fig. 7. Chromatin feature accessibility around Gad1 and Slc32a1 genes in the GABAergic clusters. Supplementary Table 1. Genomic position of ATAC features. Supplementary Table 2. Single-cell sample statistics. Supplementary Table 3. Neurotransmitter identity phenotyping. Supplementary Table 4. Cell counts per brain region and neurotransmitter phenotype. Supplementary Table 5. Unique marker gene combinations for the scATAC-seq clusters. Supplementary Table 6. Clustering resolution optimization. Supplementary Table 7. Biological function of genes associated with differentially accessible chromatin between neighboring clusters in pseudotime. Supplementary Table 8. Comparison of scATAC and scRNA clusters. [file 12864_2023_9836_MOESM1_ESM.zip › Supplementary Material_141123.pdf]

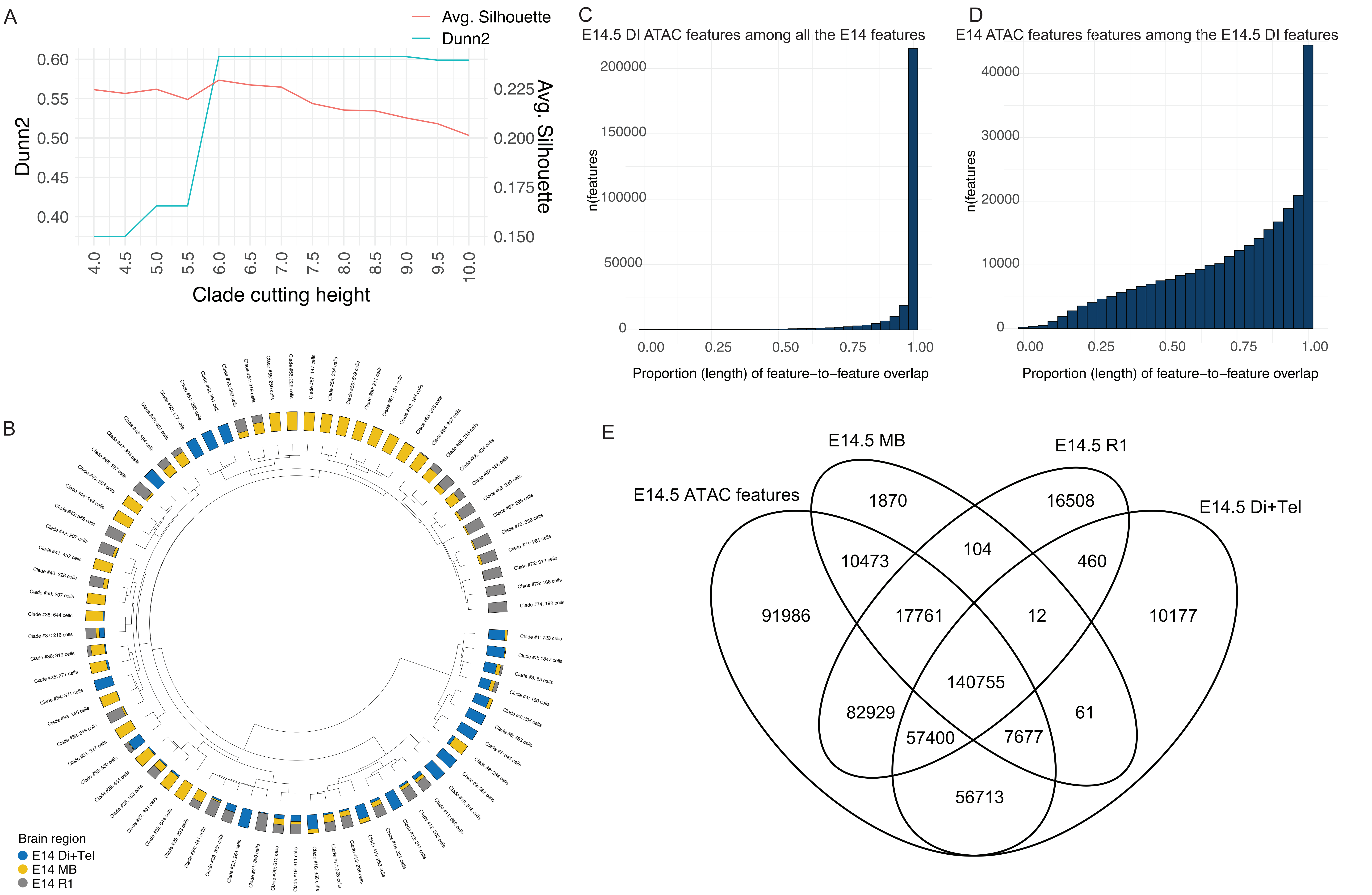
A
Avg. Silhouette
Dunn2
0.60
0.55
0.50
0.45
0.40
Dunn2
0.225
0.200
0.175
0.150
Avg. Silhouette
4.0
4.5
5.0
5.5
6.0
6.5
7.0
7.5
8.0
8.5
9.0
9.5
10.0
Clade cutting height
C
E14.5 DI ATAC features among all the E14 features
200000
150000
100000
50000
0
n(features
0.00
0.25
0.50
0.75
1.00
Proportion (length) of feature−to−feature overlap
D
E14 ATAC features features among the E14.5 DI features
40000
30000
20000
10000
0
n(features
0.00
0.25
0.50
0.75
1.00
Proportion (length) of feature−to−feature overlap
B
Brain region
E14 Di+Tel
E14 MB
E14 R1
E
E14.5 MB
E14.5 R1
E14.5 ATAC features
E14.5 Di+Tel
1870
16508
104
10473
460
91986
17761
12
10177
140755
82929
61
57400
7677
56713

Supplementary Figure 1. Distribution of cells in clades and the comparison of the features defined from individual scATAC-seq samples with the joined feature space.
A. Dunn2 statistic (green) and cluster average Silhouette statistic (red) for each configuration of clusters acquired by cutting cellular tree from heights 4 to 10, with 0.5 step. The method seeks for maximum of both values, and minimal cutting height where distinct cell populations are separated.
B. Dendrogram of cellular clades after applying the cutting height 6. Stacked barplot in second track shows the proportion of cells derived from each brain region. Outermost track shows the clade numbers and the number of cells per clade.
C. Histogram of accessible features in the E14.5 DI sample that overlap with the features in E14.5 feature space, stratified by the proportion of overlap.
D. Histogram of E14.5 features that overlap with the features defined using only E14.5 DI (E14.5 DI features), stratified by the proportion of overlap.
E. Venn diagram of feature correspondences between separately defined and the E14.5 ATAC features.

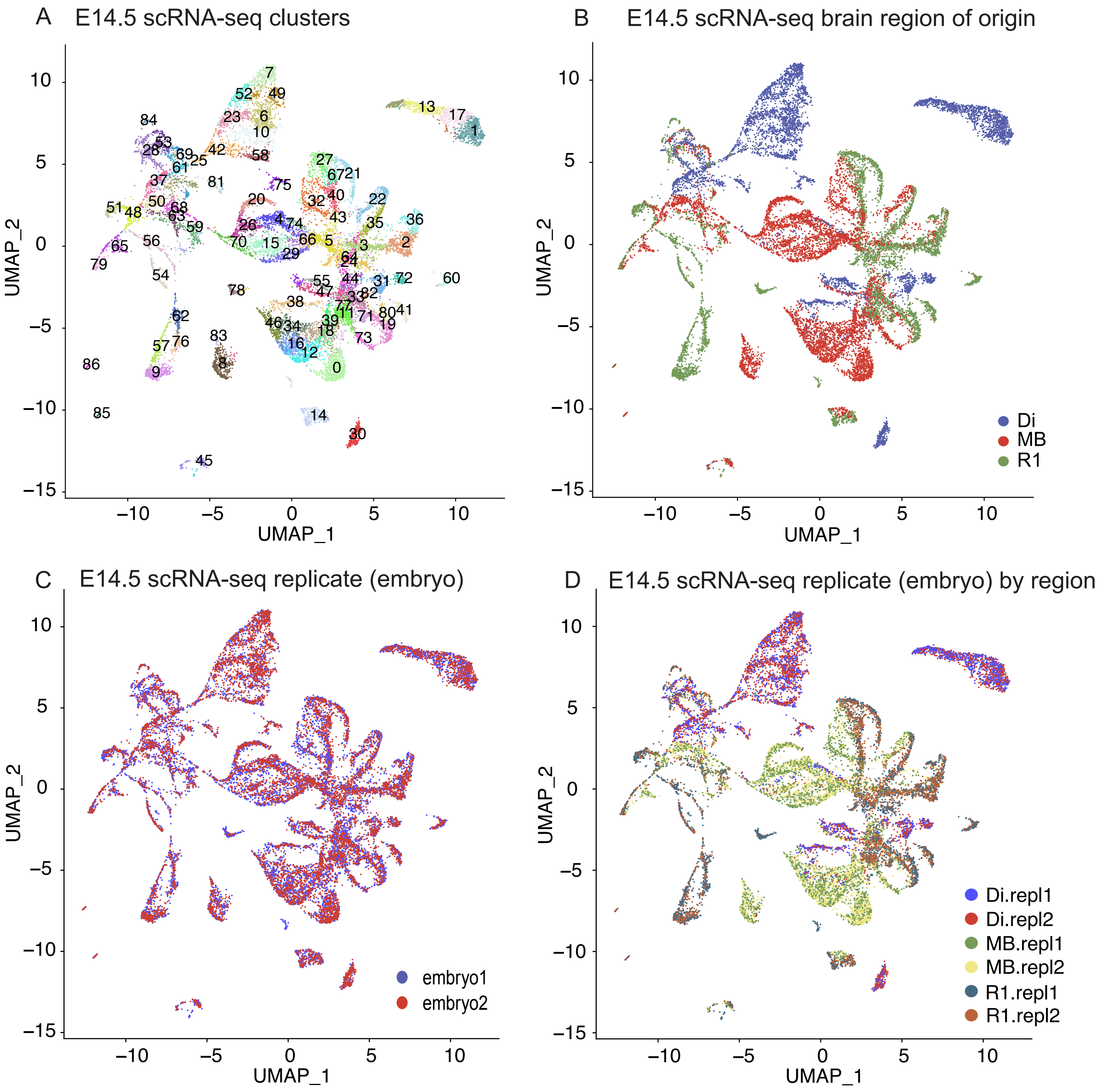
A
E14.5 scRNA-seq clusters
UMAP_1
UMAP_2
B
E14.5 scRNA-seq brain region of origin
Di
MB
R1
UMAP_1
UMAP_2
C
E14.5 scRNA-seq replicate (embryo)
embryo1
embryo2
UMAP_1
UMAP_2
D
E14.5 scRNA-seq replicate (embryo) by region
Di.repl1
Di.repl2
MB.repl1
MB.repl2
R1.repl1
R1.repl2
UMAP_1
UMAP_2

Supplementary Figure 2. Batch effect and quality control analysis of E14.5 scRNA-seq samples
A. UMAP projection of clusters (n=87) of E14.5 scRNA-seq data. B. UMAP projection of clusters, cells colored based on brain region of origin. C. UMAP of scRNA-seq clusters, cells colored based on the replicate (embryo). C. UMAP of scRNA-seq clusters, cells colored based on replicate/embryo and region of origin.

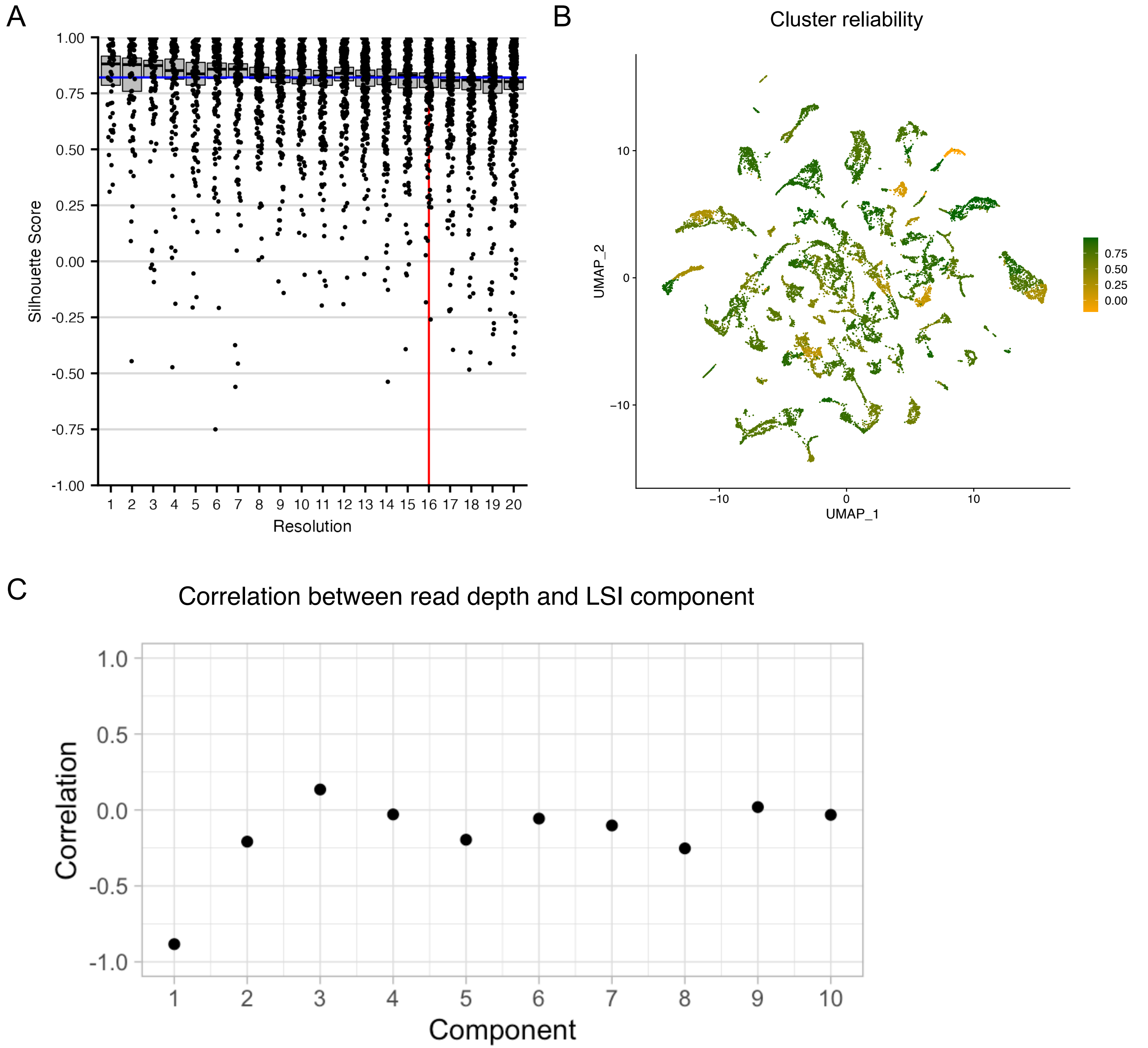

A
Silhouette Score
Resolution
1.00
0.75
0.50
0.25
0.00
-0.25
-0.50
-0.75
-1.00
1 2 3 4 5 6 7 8 9 10 11 12 13 14 15 16 17 18 19 20
B
Cluster reliability
UMAP_2
UMAP_1
10
0
−10
0.75
0.50
0.25
0.00
C
Correlation between read depth and LSI component
Correlation
Component
1.0
0.5
0.0
-0.5
-1.0
1 2 3 4 5 6 7 8 9 10

Supplementary Figure 3. Clustering resolution, cluster reliability and the similarity of labels after the scRNA and scATAC based clustering.

A. Silhouette statistic scores per clustering resolution calculated using *chooseR*. Optimal resolution is indicated with red line. Each dot represents at cluster. See the Supplementary Table 6 for the number of clusters at each resolution from res 1-20 and other details.

B. Silhouette scores per cluster overlayed on the UMAP projection of clusters. Higher values (max 1) indicate clusters with high reliability. In further analysis, the representativeness of cell types should be carefully considered for clusters with the average silhouette score of <0.5.

C. Correlation between read depth and LSI component. High (r<-0.5 or r>0.5) positive or negative correlation indicates components to be excluded from further analysis.

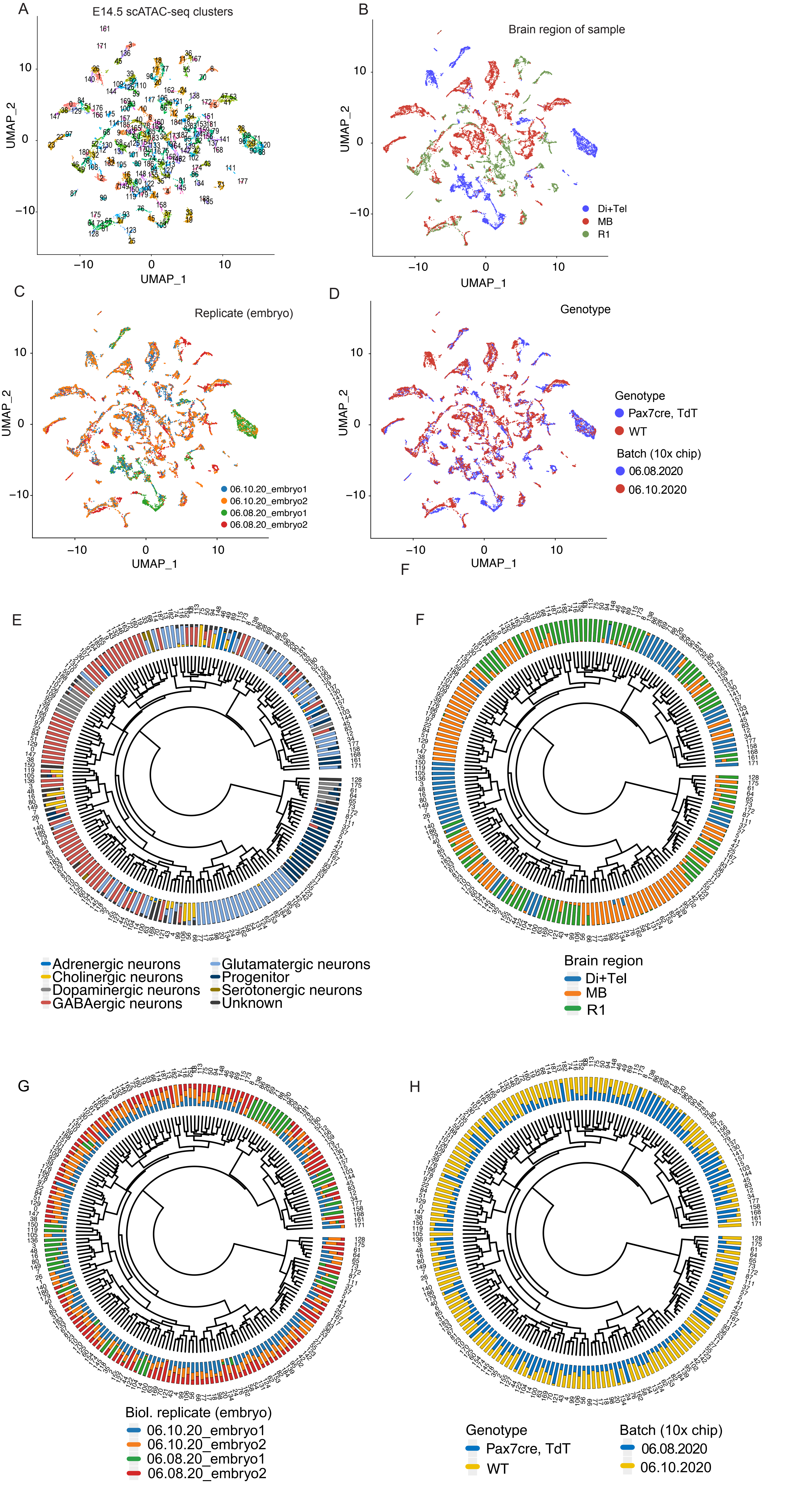
A
E14.5 scATAC-seq clusters
UMAP_1
UMAP_2
B
Brain region of sample
Di+Tel
MB
R1
C
Replicate (embryo)
06.10.20_embryo1
06.10.20_embryo2
06.08.20_embryo1
06.08.20_embryo2
D
Genotype
Pax7cre, TdT
WT
Batch (10x chip)
06.08.2020
06.10.2020
F
E
Adrenergic neurons
Cholinergic neurons
Dopaminergic neurons
GABAergic neurons
Glutamatergic neurons
Progenitor
Serotonergic neurons
Unknown
F
Brain region
Di+Tel
MB
R1
G
Biol. replicate (embryo)
06.10.20_embryo1
06.10.20_embryo2
06.08.20_embryo1
06.08.20_embryo2
H
Genotype
Pax7cre, TdT
WT
Batch (10x chip)
06.08.2020
06.10.2020

Supplementary Figure 4. Batch effect and quality control analysis of scATAC-seq samples

A. E14.5 scATAC-seq clusters (n=189) on UMAP. B. Cells on the UMAP colored based on brain region of origin. C. Cells on the UMAP colored based on replicate (embryo). D. Cells on the UMAP colored based on genotype of mouse. E. Circosplot of all E14.5 scATAC-seq clusters (same as Fig. 5), with the proportion of cells in each cluster by the NT-type shown as additional track. F. Circosplot of all E14.5 scATAC-seq clusters, with brain region of origin proportions shown. G. Circosplot of all E14.5 scATAC-seq clusters, with replicate (embryo) proportions shown. H. Circosplot of all E14.5 scATAC-seq clusters, with genotype proportions shown.

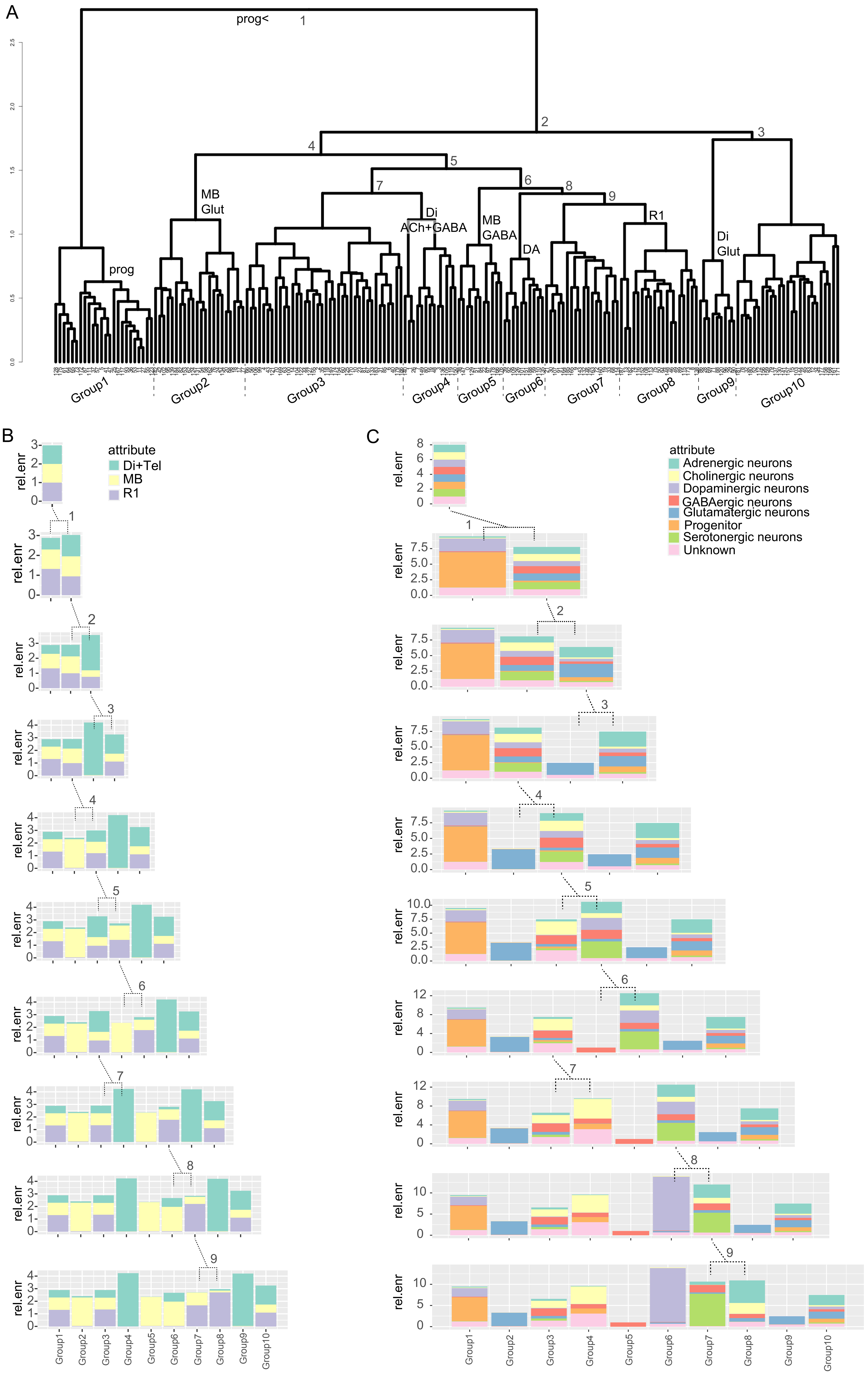
A
prog<
1
2
3
4
5
6
7
8
9
MB
Glut
Di
ACh+GABA
MB
GABA
DA
R1
Di
Glut
prog
Group1
Group2
Group3
Group4
Group5
Group6
Group7
Group8
Group9
Group10
B
rel.enr
attribute
Di+Tel
MB
R1
C
attribute
Adrenergic neurons
Cholinergic neurons
Dopaminergic neurons
GABAergic neurons
Glutamatergic neurons
Progenitor
Serotonergic neurons
Unknown

Supplementary Figure 5. Analysis of the hierarchical tree of cell clusters.

A. Hierarchical tree of scATAC-seq clusters (also shown in Figure 5). The first 10 branching levels are indicated in numbers at the point of new branch appearing. The brain region or NT-type label is shown for the cluster groups of uniform brain region or NT-type identity, at the first appearance of the cluster group.

B. Relative enrichment (rel.enr) of cells originating from the different brain region samples at each indicated branch of the clusters tree. Relative enrichment is shown for the first 10 branching levels. rel.enr = 1 equals no enrichment of attribute between the groups in the given branching level, and any deviation from 1 shows either increase or decrease in relative proportion of the attribute (See Methods). The cell groups at the the brancing level 10 (Group 1 - Group 10) are shown in the tree in (A) and below the last plots in (B) and (C).

C. Relative enrichment of NT-type categories in the cell groups at the first 10 branching levels.

NT, neurotransmitter type; reg, brain region identity. Brain regions: Di+Tel, diencephalon and telencephalon samples; MB, midbrain samples; R1, rhombomere 1 samples. ACh+GABA, GABA- and acetylcholinergic dual neurotransmitter neurons; GLUT, glutamatergic neurons; GABA, GABAergic neurons; DA, dopaminergic neurons; prog, progenitor cells.

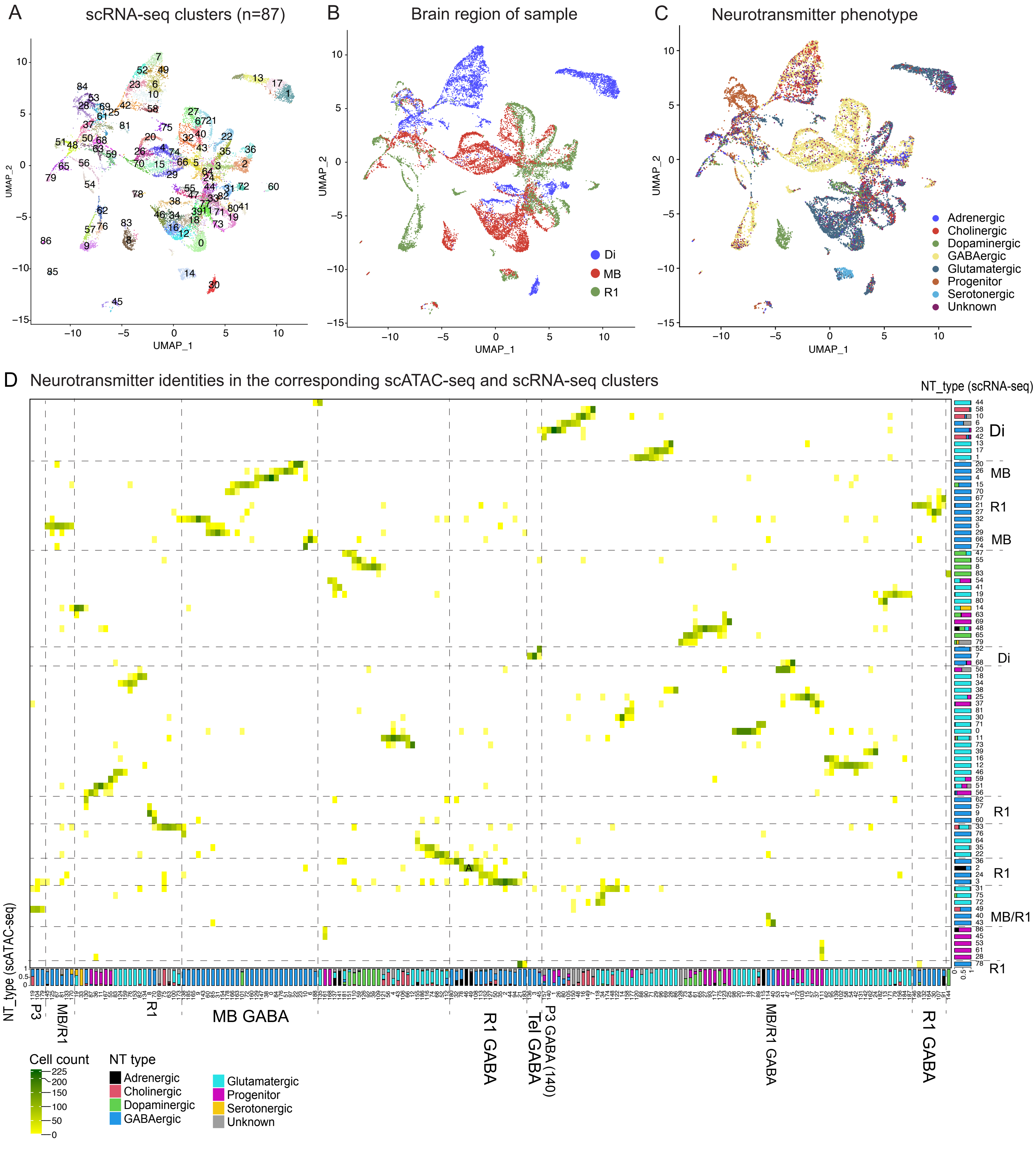

A
scRNA-seq clusters (n=87)
B
Brain region of sample
C
Neurotransmitter phenotype
UMAP_1
UMAP_2
Di
MB
R1
Adrenergic
Cholinergic
Dopaminergic
GABAergic
Glutamatergic
Progenitor
Serotonergic
Unknown
D
Neurotransmitter identities in the corresponding scATAC-seq and scRNA-seq clusters
NT_type (scRNA-seq)
NT_type (scATAC-seq)
P3
MB/R1
MB GABA
R1 GABA
Tel GABA
P3 GABA (140)
MB/R1 GABA
R1 GABA
Cell count
NT type

Supplementary Figure 6. Clustering and neurotransmitter phenotype annotation of E14.5 DI, MB and R1 scRNA-seq data.

A. UMAP of the 87 scRNA-seq clusters, at the optimal clustering resolution according to chooseR.

B. The scRNA-seq cluster UMAP, with brain region shown for each cell.

C. The scRNA-seq cluster UMAP, with neurotransmitter phenotype (NT-type) labels shown.

D. Heatmap of cluster-to-cluster matches between the integrated and scATAC-seq based (columns) and scRNA-seq based (rows) clusters. The stacked barplots show the proportion of cells of each neurotansmitter type in the cluster. Matching clusters mostly received the same NT-type label and originate from the same brain region. Matching adrenergic neuron (A) clusters 2 (scRNA) vs 46, 49 (scATAC) are grouped with R1 GABAergic neurons.

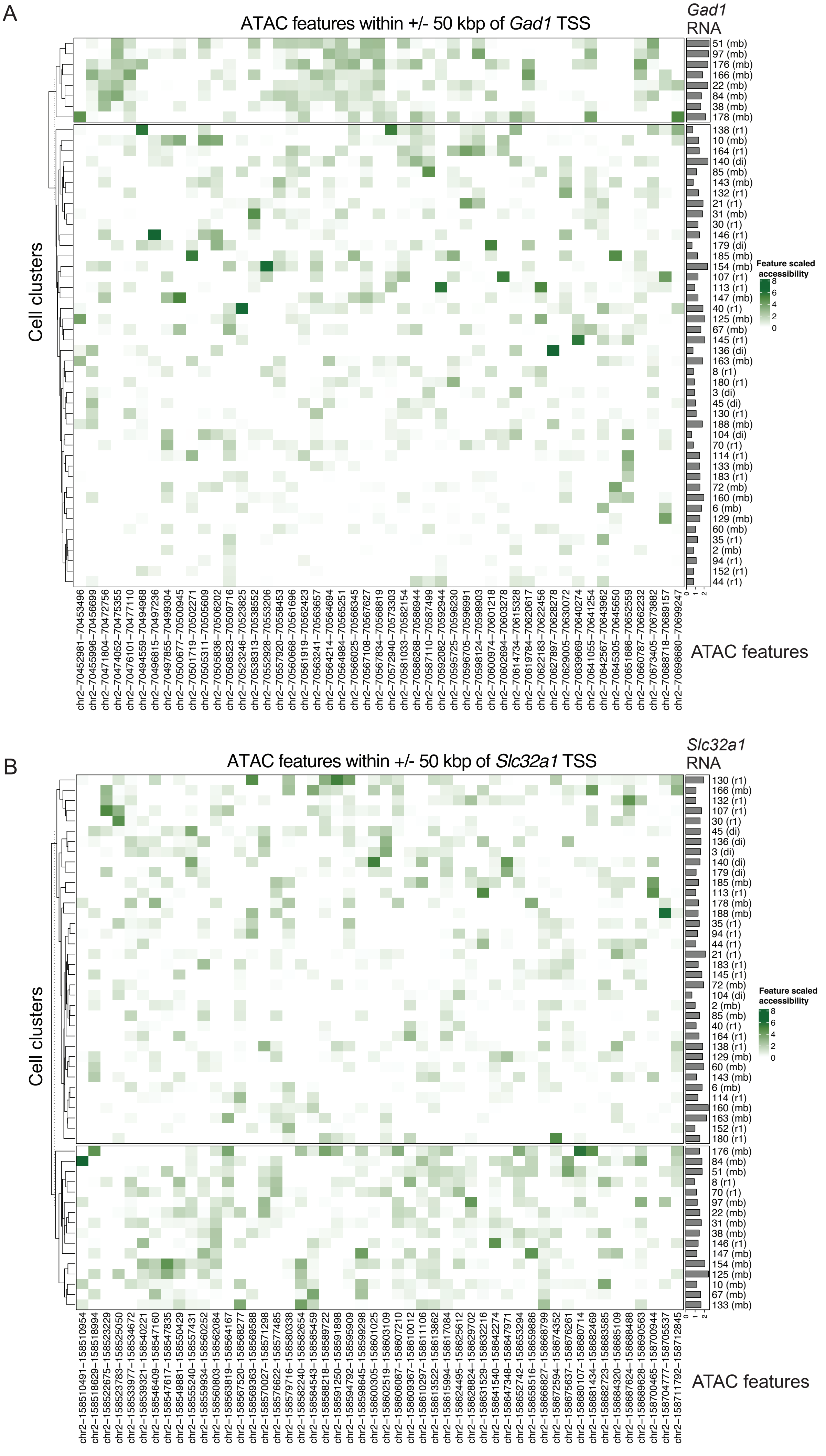

A
ATAC features within +/- 50 kbp of Gad1 TSS
Gad1
RNA
Cell clusters
51 (mb)
97 (mb)
176 (mb)
166 (mb)
22 (mb)
84 (mb)
38 (mb)
178 (mb)
138 (r1)
10 (mb)
164 (r1)
140 (di)
85 (mb)
143 (mb)
132 (r1)
21 (r1)
31 (mb)
30 (r1)
146 (r1)
179 (di)
185 (mb)
154 (mb)
107 (r1)
113 (r1)
147 (mb)
40 (r1)
125 (mb)
67 (mb)
145 (r1)
136 (di)
163 (mb)
8 (r1)
180 (r1)
3 (di)
45 (di)
130 (r1)
188 (mb)
104 (di)
70 (r1)
114 (r1)
133 (mb)
183 (r1)
72 (mb)
160 (mb)
6 (mb)
129 (mb)
60 (mb)
35 (r1)
2 (mb)
94 (r1)
152 (r1)
44 (r1)
Feature scaled accessibility
8
6
4
2
0
0 1 2
ATAC features
chr2−70452981−70453496
chr2−70455996−70456699
chr2−70471804−70472756
chr2−70474052−70475355
chr2−70476101−70477110
chr2−70494559−70494968
chr2−70496815−70497236
chr2−70497855−70499304
chr2−70500677−70500945
chr2−70501719−70502271
chr2−70505311−70505609
chr2−70505836−70506202
chr2−70508523−70509716
chr2−70523246−70523825
chr2−70538313−70538552
chr2−70552928−70553206
chr2−70557920−70558453
chr2−70560668−70561696
chr2−70561919−70562423
chr2−70563241−70563657
chr2−70564214−70564694
chr2−70564984−70565251
chr2−70566025−70566345
chr2−70567108−70567627
chr2−70567834−70568819
chr2−70572940−70573303
chr2−70581033−70582154
chr2−70586268−70586944
chr2−70587110−70587499
chr2−70592082−70592944
chr2−70595725−70596230
chr2−70596705−70596991
chr2−70598124−70598903
chr2−70600974−70601218
chr2−70602694−70603278
chr2−70614734−70615328
chr2−70619784−70620617
chr2−70622183−70622456
chr2−70627897−70628278
chr2−70629005−70630072
chr2−70639669−70640274
chr2−70641055−70641254
chr2−70642567−70643962
chr2−70645305−70645650
chr2−70651686−70652559
chr2−70660787−70662232
chr2−70673405−70673882
chr2−70688718−70689157
chr2−70698680−70699247
B
ATAC features within +/- 50 kbp of Slc32a1 TSS
Slc32a1
RNA
Cell clusters
130 (r1)
166 (mb)
132 (r1)
107 (r1)
30 (r1)
45 (di)
136 (di)
3 (di)
140 (di)
179 (di)
185 (mb)
113 (r1)
178 (mb)
188 (mb)
35 (r1)
94 (r1)
44 (r1)
21 (r1)
183 (r1)
145 (r1)
72 (mb)
104 (di)
2 (mb)
85 (mb)
40 (r1)
164 (r1)
138 (r1)
129 (mb)
60 (mb)
143 (mb)
6 (mb)
114 (r1)
160 (mb)
163 (mb)
152 (r1)
180 (r1)
176 (mb)
84 (mb)
51 (mb)
8 (r1)
70 (r1)
97 (mb)
22 (mb)
31 (mb)
38 (mb)
146 (r1)
147 (mb)
154 (mb)
125 (mb)
10 (mb)
67 (mb)
133 (mb)
Feature scaled accessibility
8
6
4
2
0
0 1 2
ATAC features
chr2−158510491−158510954
chr2−158518629−158518994
chr2−158522675−158523229
chr2−158523783−158525050
chr2−158533977−158534672
chr2−158539321−158540221
chr2−158546409−158547160
chr2−158547617−158547835
chr2−158549881−158550429
chr2−158555240−158557431
chr2−158559934−158560252
chr2−158560803−158562084
chr2−158563819−158564167
chr2−158567520−158568277
chr2−158569363−158569588
chr2−158570027−158571298
chr2−158576622−158577485
chr2−158579716−158580338
chr2−158582240−158582654
chr2−158584543−158585459
chr2−158588218−158589722
chr2−158591250−158591898
chr2−158594792−158595909
chr2−158598645−158599298
chr2−158600305−158601025
chr2−158602519−158603109
chr2−158606087−158607210
chr2−158609367−158610012
chr2−158610297−158611106
chr2−158613522−158613862
chr2−158615994−158617084
chr2−158624495−158625612
chr2−158628824−158629702
chr2−158631529−158632216
chr2−158641540−158642274
chr2−158647348−158647971
chr2−158652742−158653294
chr2−158658516−158659886
chr2−158666827−158668799
chr2−158672594−158674352
chr2−158675637−158676261
chr2−158680107−158680714
chr2−158681434−158682469
chr2−158682723−158683585
chr2−158684320−158685109
chr2−158687624−158688488
chr2−158689628−158690563
chr2−158700465−158700944
chr2−158704777−158705537
chr2−158711792−158712845

Supplementary Figure 7. Chromatin feature accessibility around Gad1 and Slc32a1 genes in the GABAergic clusters

A Heatmap of the accessibility of ATAC features +/-50kpb around *Gad1* gene, one of the strongest indicator genes for GABAergic fate, across GABAergic clusters (rows). Dominant brain region of origin is shown in parenthesis after the cluster number. Barplots on the right side show average expression of *Gad1* in the clusters. Accessibility data has been column scaled and rows are clustered with Euclidean as distance and ward.D2 as linkage method. B. Accessibility of ATAC features within +/-50kpb of *Slc32a1* gene and the *Slc32a1* RNA expression in the GABAergic cell clusters, similar to (A).

Supplementary Table legends

Supplementary Table 1. Genomic position of ATAC features.
Comparison of the position of the E14.5 ATAC features defined herein with randomised genomic position.

Supplementary Table 2. Single-cell sample statistics.
Single-cell RNA-seq and single-cell ATAC-seq sample statistics. The method is abbreviated in the table as ATAC, scATAC-seq or RNA, scRNA-seq. For scATAC-seq samples, the embryonic stage (E14.5), brain region of sample, genotype of the embryo, number of cells per sample, median number of fragments per cell, minimum number of fragments per barcode required to assign the barcode to a cell, the percent of reads from the sample included in final merged data, the sample name used in the SRA repository, the sample collection date (batch) and the median of ratios of fragments per cell included in the in E14.5 features is shown. For scRNA-seq samples, the embryonic stage (E14.5), brain region of sample, genotype of the embryo, number of cells per sample, median number of reads per cell, median number of genes detected in the cells, the percent of reads from the sample included in final merged data, the sample name used in the SRA repository, the sample collection date (batch) is shown.

Supplementary Table 3. Neurotransmitter identity phenotyping.
Sheet 1: ScType database for assigning the NT type labels.
Sheet 2: CellCycleScore genes expressed in the E14.5 mouse neuronal progenitors and list of genes used to define the *g2m* and *s* artificial genes in ScType.

Supplementary Table 4. Cell counts per brain region and neurotransmitter phenotype.
Identities were assigned per cell. The cluster was counted in category when >50% of cells in the cluster match the category.

Supplementary Table 5. Unique marker gene combinations for the scATAC-seq clusters.
Table listing the scATAC-seq cell clusters (n=189), the unique marker gene combination for each cluster, calculated using CombiROC (marker.combination), the main neurotransmitter type in the cluster (top_nt_type) and main brain region identity in the cluster (top_brain_region). The top_nt_type and top_brain_region label was assigned if >50% of the cells in the cluster receive the label, otherwise NA. The clusters labelled NA may to contain several cell types, or migratory cells found in neighboring brain regions.

Supplementary Table 6. Clustering resolution optimization.
Results of *ChooseR* iteration for finding optimal resolution of clustering the integrated single-cell dataset based on accessible chromatin features. The clustering was iterated from res=1 to res=20.

Supplementary Table 7. Biological function of genes associated with differentially accessible chromatin between neighboring clusters in pseudotime.
Analysis of Genome Ontology term enrichment in genes near differentially accessible chromatin features between cluster pairs. Selected clusters represent lineage related telencephalic and diencephalic GABAergic neurons.

Supplementary Table 8. Comparison of scATAC and scRNA clusters.
Contingency table of cell placement in scATAC clusters and scRNA clusters.

Additional File at Github. Analysis of the *CombiROC* cluster marker gene expressions.
Available at
https://github.com/ComputationalNeurogenetics/NeuronalFeatureSpace/blob/main/analysis/Combiroc_avg_plots110923_comp.pdf
